# Prevalence of chronic cough and possible causes in the general population based on the Korean National Health and Nutrition Examination Survey

**DOI:** 10.1097/MD.0000000000004595

**Published:** 2016-09-16

**Authors:** Hyeon-Kyoung Koo, Ina Jeong, Sei Won Lee, Jinkyeong Park, Joo-Hee Kim, So Young Park, Hye Yun Park, Chin Kook Rhee, Yee Hyung Kim, Ji Ye Jung, Sung-Kyoung Kim, Yong Hyun Kim, Eun Young Choi, Ji-Yong Moon, Jong-Wook Shin, Jin Woo Kim, Kyung Hoon Min, Sei Won Kim, Kwang Ha Yoo, Je Hyeong Kim, Seung Hun Jang, Hyoung Kyu Yoon, Hui Jung Kim, Ki-Suck Jung, Deog Kyeom Kim

**Affiliations:** a Divison of Pulmonary and Critical Care Medicine, Department of Internal Medicine, Ilsan Paik Hospital, Inje University College of Medicine, Ilsan; b Department of Internal Medicine, National Medical Center, Seoul; c Department of Pulmonology and Critical Care Medicine, Asan Medical Center, University of Ulsan College of Medicine, Seoul; d Department of Critical Care, Samsung Medical Center, Sungkyunkwan University School of Medicine, Seoul; e Division of Pulmonary, Allergy, and Critical Care Medicine, Department of Medicine, Hallym University Sacred Heart Hospital, Hallym University College of Medicine, Anyang; f Division of Pulmonary and Critical Care Medicine, Kyung Hee Medical Center, Kyung Hee University; g Division of Pulmonary and Critical Care Medicine, Department of Medicine, Samsung Medical Center, Sungkyunkwan University School of Medicine, Seoul; h Division of Pulmonary, Allergy and Critical Care Medicine, Department of Internal Medicine, Seoul St Mary's Hospital, College of Medicine, The Catholic University of Korea, Seoul; i Department of Pulmonary and Critical Care Medicine, Kyung Hee University Hospital at Gangdong, Kyung Hee University, Seoul; j Division of Pulmonary, Department of Internal Medicine, Severance Hospital, Yonsei University College of Medicine, Seoul; k Division of Pulmonology, Department of Internal Medicine, St. Vincent's Hospital, College of Medicine, The Catholic University of Korea, Suwon; l Division of Pulmonary, Allergy and Critical Care Medicine, Department of Internal Medicine, Bucheon St. Mary's Hospital, College of Medicine, The Catholic University of Korea, Bucheon; m Department of Pulmonary and Allergy, Department of Internal Medicine, Regional Respiratory Center, Yeungnam University Hospital, Daegu; n Department of Internal Medicine, Hanyang University College of Medicine, Seoul, Korea; o Division of Pulmonary, Allergy, and Critical Care Medicine, Department of Internal Medicine, Chung-Ang University College of Medicine, Seoul; p Division of Pulmonology and Critical Care Medicine, Department of Internal Medicine, Uijeongbu St Mary's Hospital, College of Medicine, The Catholic University of Korea, Uijeongbu; q Division of Pulmonary, Sleep and Critical Care Medicine, Department of Internal Medicine, Korea University Guro Hospital, Korea University Medical School, Seoul; r Division of Pulmonary and Critical Care Medicine, Department of Internal Medicine, Yeouido St Mary's Hospital, College of Medicine, The Catholic University of Korea, Seoul; s Division of Pulmonary, Allergy and Critical Care Medicine, Department of Internal Medicine, KonKuk University School of Medicine, Seoul; t Division of Pulmonary and Critical Care Medicine, Department of Internal Medicine, Korea University Ansan Hospital, Korea University College of Medicine, Ansan; u Division of Pulmonary and Critical Care Medicine, Department of Internal Medicine, Wonkwang University Sanbon Hospital, Wonkwang University College of Medicine, Sanbon; v Division of Pulmonary and Critical Care Medicine, Department of Internal Medicine, Seoul Metropolitan Government-Seoul National University Boramae Medical Center, Seoul National University College of Medicine, Seoul, Republic of Korea.

**Keywords:** chronic cough, COPD, KNHANES, prevalence, smoking, upper airway cough syndrome

## Abstract

Although chronic cough is very common, its prevalence and causes have been rarely reported in the large general population including smokers. This study aimed to identify the prevalence of possible causes of chronic cough and their clinical impact.

From Korean National Health and Nutrition Examination Survey (KNHANES) data including 119,280 adults aged over 40 years, 302 individuals with chronic cough were recruited irrespective of smoking status. Data from questionnaire, laboratory tests including spirometry, chest radiographs, and otorhinolaryngologic examination were analyzed.

The prevalence of chronic cough in adults was 2.5% ± 0.2%. Current smokers occupied 47.7% ± 3.8% of study population and 46.8% ± 3.9% of the subjects showed upper airway cough syndrome (UACS). Based on spirometry, chronic obstructive pulmonary disease (COPD) was identified in 26.4% ± 3.5%. Asthma explained for 14.5% ± 2.8% of chronic cough. Only 4.1% ± 1.6% showed chronic laryngitis suggesting gastro-esophageal reflux-related cough. Abnormalities on chest radiography were found in 4.0% ± 1.2%. Interestingly, 50.3% ± 4.5% of study subjects had coexisting causes. In multivariate analysis, only current smoking (odds ratio [OR] 3.16, *P* < 0.001), UACS (OR 2.50, *P* < 0.001), COPD (OR 2.41, *P* < 0.001), asthma (OR 8.89, *P* < 0.001), and chest radiographic abnormalities (OR 2.74, *P* = 0.003) were independent risk factor for chronic cough. This pattern was not different according to smoking status excepting the prevalence of COPD.

Smoking, COPD, and chest radiographic abnormalities should be considered as causes of chronic cough, along with UACS and asthma. Gastro-esophageal reflux-related cough is not prevalent in study population.

## Abstract 2

Supplemental Digital Content is available in the text

## 1 Introduction

Cough is an important defense mechanism in the airway and a common symptom of many pulmonary and extra-pulmonary diseases.^[1,2]^ However, for many patients, cough may be regarded merely as an annoyance, particularly if chronic.^[3–5]^

Most guidelines on chronic cough emphasize the upper airway cough syndrome (UACS), asthma, and gastro-esophageal reflux disease (GERD) as usual causes of chronic cough in nonsmokers with normal chest radiographs.^[6–13]^ However, the data in these recommendations were reported a long time ago.^[3–11]^ Further, the prevalence of diseases that cause chronic cough, such as asthma, GERD, and other comorbidities, differs according to region and ethnicity.^[14]^ The prevalence of asthma is higher in urbanized communities adopting a Western lifestyle^[15]^; notably, the prevalence of GERD is reported to be 10% to 20% in the Western area but less than 5% in Asia.^[16]^

In clinical practice, a significant number of smokers complain of chronic cough; therefore, many clinicians have questions about the actual prevalence and clinical characteristics of diseases contributing to chronic cough. Nevertheless, reports on the possible causes of chronic cough are not up to date and any relevant data come from relatively small populations. Additionally, the prevalence study of chronic cough has been rarely reported in the large general population including smokers. Updated research on the prevalence of chronic cough and the impact that various conditions have on it is now mandatory. Moreover, many diseases affecting the respiratory tract show environmental, regional, and ethnic differences, and the same might apply to causes of chronic cough.

Recently published Korean guideline listed the various causes of chronic cough, placing emphasis on the major ones.^[17]^ However, there are still little data on the prevalence of possible causes or the impact of each diseases on chronic cough in general population including smokers.

This study aimed to identify the prevalence of chronic cough and its possible causes, along with the relative impact of each cause on the prevalence of cough in the general population using data from the Korean National Health and Nutrition Examination Survey (KNHANES).

## 2 Methods

### 2.1 Study population

The KNHANES is a collection of nationally representative, cross-sectional, population-based health, and nutritional surveys produced by the Korean Centers for Disease Control and Prevention.^[18]^ Briefly, participants in KNHANES were chosen by proportional allocation sampling with multistage stratification, based on geography, age, and sex. KNHANES includes a health interview, physical examination, laboratory tests, and nutritional questionnaires to assess the health and nutritional status of the noninstitutionalized civilian population of Korea. The health interview included an established questionnaire to determine the demographic and socioeconomic characteristics of the subjects including age, education level, occupation, income, marital status, smoking habits, alcohol consumption, exercise, past and current diseases, and family history. A field survey team including otorhinolaryngologists performed the interviews and physical examinations in a mobile examination unit. All individuals participated voluntarily and provided their written informed consent. The KNAHENS protocol was approved by the Korean Centers for Disease Control and Prevention institutional review board.

### 2.2 Measurements

Spirometry was performed for subjects aged >40 years according to the guidelines of the American Thoracic Society/European Respiratory Society,^[19]^ using a spirometry system (model 1022; SensorMedics Corporation San Diego, CA). Predicted values were calculated using the predictive equation for the Korean population.^[20]^ Chest radiographs were evaluated and interpreted by a pulmonologist and a radiologist. Quality of life was measured using a validated Korean version of the 5-item self-administered EuroQOL (EQ-5D).^[21]^ An otorhinolaryngologic examinations were performed by trained otorhinolaryngologists according to standardized protocols. Examinations of the nasal cavity were performed using a 4 mm, 0° nasal endoscope before and after decongestion. Laryngoscopic vocal cord examinations were performed using a 4 mm 70° angled rigid endoscope with a CCD camera. The Epidemiologic Survey Committee of the Korean Otorhinolaryngologic Society prepared a protocol for the diagnosis of chronic laryngitis. This committee verified the quality of the survey by periodically visiting the mobile examination units, educating participating doctors, obtaining laryngeal examination data, and data-proofing using video documentation of the larynx throughout the study. Two otorhinolaryngologic surgeons from the Korean Otorhinolaryngologic Society subsequently confirmed the video documentation and assessed the disease decision protocol. Documentation of the video was obtained as 640 × 480-sized Audio Video Interleave files, which were compressed by DivX 4.12 codec using a compression rate of 6 Mb/s.

### 2.3 Definitions

Rhinitis and chronic sinusitis were diagnosed according to standardized protocols. If symptoms or physical examination indicated rhinitis or chronic sinusitis, UACS was diagnosed. Laryngoscopic findings of laryngitis and/or inflammation, including Reinke edema, pseudosulcus, erythema, or thick endolaryngeal mucus, were diagnosed as chronic laryngitis.

Chronic obstructive pulmonary disease (COPD) was defined as a spirometric result for forced expiratory volume in 1 second/forced vital capacity of <0.7 in adults aged >40 years. A history of asthma, tuberculosis, hypertension, diabetes, hyperlipidemia, and/or cardiac disease was obtained by a self-administered questionnaire asking “Have you been diagnosed with the disease by a doctor?” or “Do you take medicine or treatment for the disease?” Since, many Koreans with COPD are misdiagnosed as having asthma, asthma was only diagnosed if the subject reported a history of asthma but did not meet the criteria for COPD.

Chest radiographic abnormalities included only conditions expected to contribute to chronic cough. Inactive lesions including healed condition or minimal extent were not regarded as abnormalities.

Hypertension was defined as blood pressure ≥140 mm Hg systolic or ≥90 mm Hg diastolic, or the use of antihypertensive medications irrespective of the blood pressure status.^[22]^ Diabetes was defined as a fasting plasma glucose level ≥126 mg/dL or hemoglobin A1c ≥6.5%, and/or a current regimen for diabetes treatment.^[23]^ Hyperlipidemia was defined as an abnormal level of high density lipoprotein (<40 mg/dL in male and <50 mg/dL in female participants), a triglyceride level >150 mg/dL, and/or currently taking lipid lowering agents.^[24]^

### 2.4 Statistical analysis

KNAHENS was designed using a complex, stratified, multistage probability sampling model, whereby the data were analyzed using the complex sample design to represent prevalence in the Korean population. The analysis was performed using SAS software (version 9.3, SAS Institute, Inc., Cary, NC).

The study population with chronic cough was compared with subjects without chronic cough. The common causes of chronic cough were described according to smoking status to identify the causes of cough in each group. The impact of each condition contributing to chronic cough was also analyzed, adjusting for age, sex, body mass index, smoking status, and comorbidities.

General linear regression was used for continuous variables and logistic regression for categorical variables. Data are shown as the mean ± standard error or as the frequency (%). A *P*-value <0.05 was considered to be statistically significant.

## 3 Results

### 3.1 Prevalence and clinical characteristics

**Figure 1:**
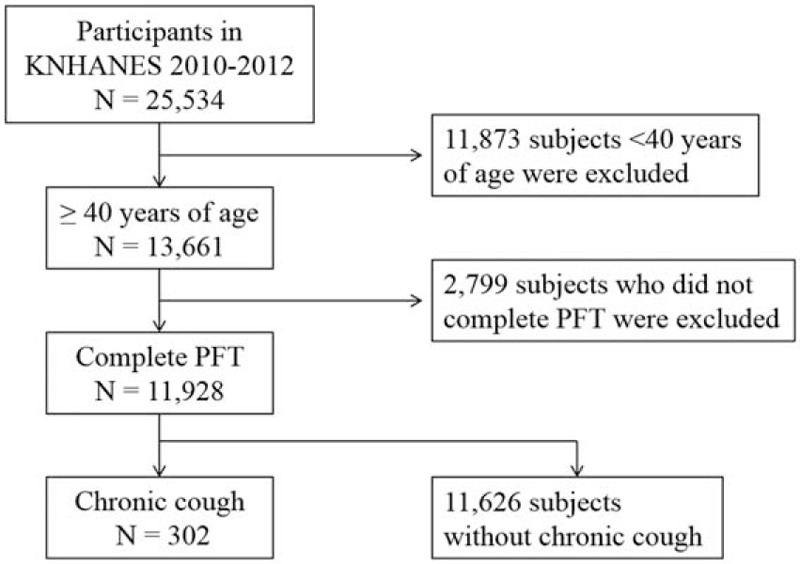
Flow chart of the study population.

Data from 11,928 adults aged >40 years who completed spirometry in 2010 to 2012 were retrieved from KNHANES. Of these, 302 had chronic cough for more than 3 months (Fig. 1). The overall prevalence of chronic cough in the Korean population aged >40 years was 2.5% ± 0.2%. When compared with people without chronic cough, those with chronic cough were older, male-predominant, and included more current smokers. Hypertension, hyperlipidemia, COPD, diabetes, history of tuberculosis, cardiac disease, asthma, and stroke were more prevalent in subjects with chronic cough. However, the prevalence of chronic laryngitis was not significantly different. The demographic and clinical characteristics of participants are summarized in Table 1.

**Table 1:**
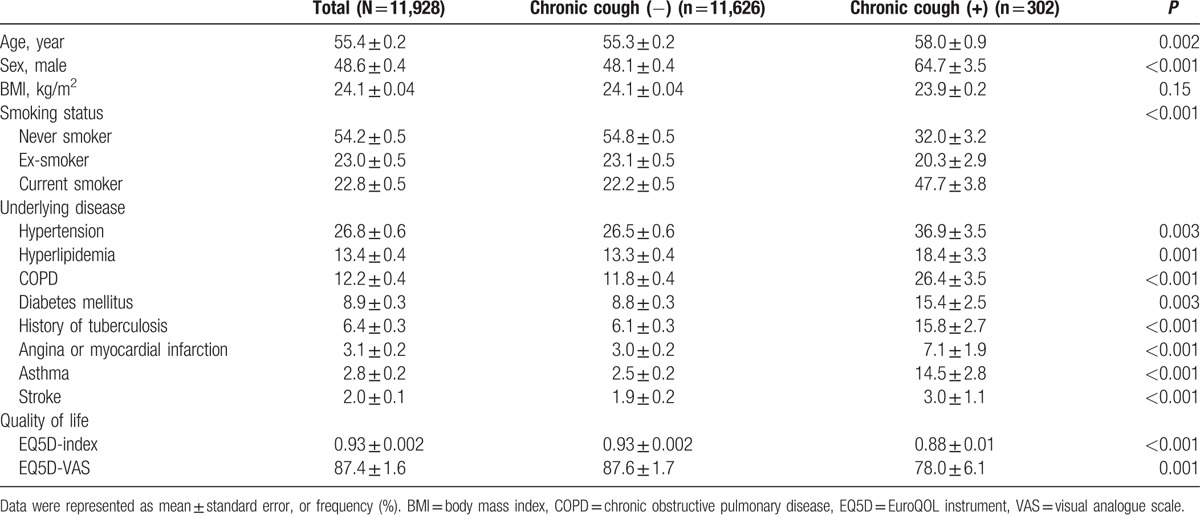
Baseline characteristics of the study population according to the presence of chronic cough.

Of the participants with chronic cough, 47.7% ± 3.8% were current smokers, 45.1% ± 3.9% had nasal symptoms, 42.2% ± 3.7% had an identifiable problem in the nasal cavity, and 46.8% ± 3.9% were suspected to UACS; 26.4 ± 3.5% were compatible with COPD by spirometry, and 14.5 ± 2.8% were compatible with asthma. Chronic laryngitis was used as an alternative to identify GERD-related cough and was presented in 4.1% ± 1.6% of the population. On chest radiography, 4.0% ± 1.2% had an abnormality implying a cause of chronic cough. Patients with chronic cough reported significantly lower quality of life (Table 1).

When the study population was stratified according to smoking status, the following factors were not significantly different between never smokers and former/current smokers: the frequencies of UACS, asthma, chronic laryngitis, abnormal chest radiographs, hypertension, diabetes, hyperlipidemia, stroke, cardiac disease, and history of tuberculosis. However, the prevalence of COPD was higher in current smokers (Table 2 and Table S1).

**Table 2:**
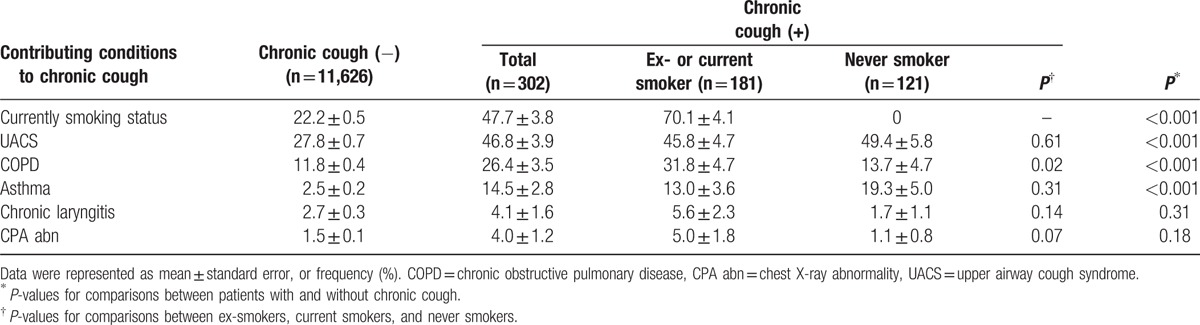
Prevalence of chronic cough-related diseases in the study population.

### 3.2 Prevalence of overlapped possible causes

**Figure 2:**
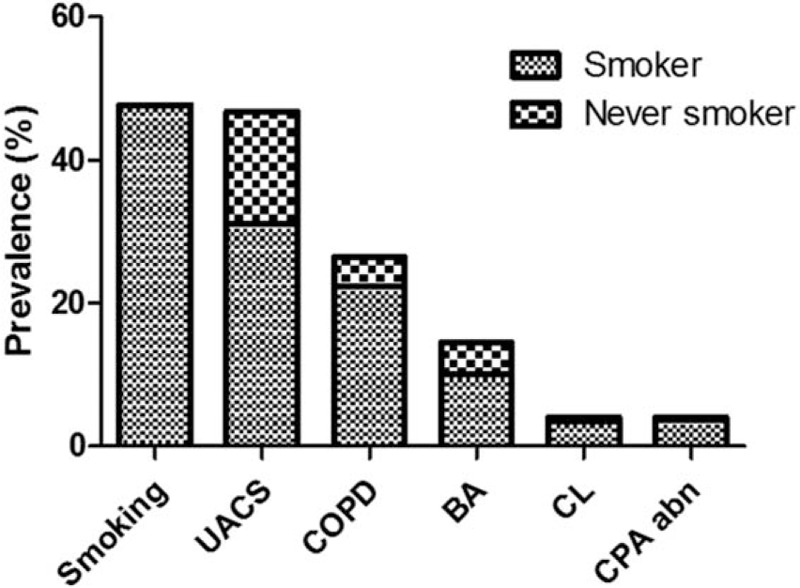
Prevalence of possible causes of chronic cough.

Given the unified airway theory that causes of chronic cough frequently coexist, we identified the frequency of coexistence of possible causes. When possible causes were classified by current smoking status, UACS, COPD, asthma, and chest radiographic abnormality, 50.3% ± 4.5% of those with chronic cough had coexisting possible causes (Fig. 2). Additionally, 12.7% ± 3.2% of those with chronic cough were suspected to have 3 or more possible causes. However, no possible causes were noted in 14.7% ± 3.1% of subjects. The most highly overlapped conditions were UACS in subjects with asthma (66.9% ± 10.8%) and chest radiographic abnormalities in those with COPD (9.9% ± 4.3%) or asthma (9.4% ± 6.3%) (Table 3).

**Table 3:**
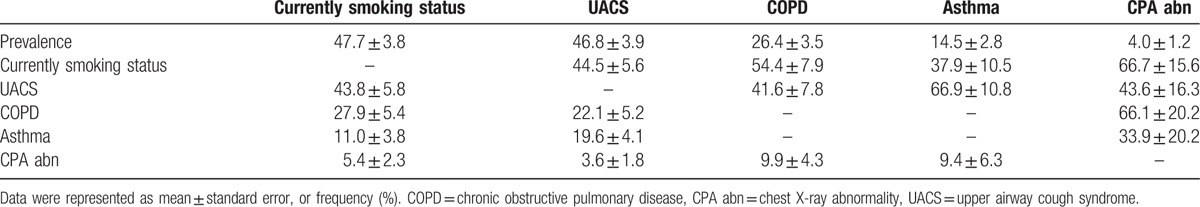
Prevalence of overlapped conditions in patients with chronic cough.

### 3.3 Relative impacts of possible causes

Since these possible causes, that is, current smoking, postnasal drip, COPD, asthma, and chest radiographic abnormality, are also observed in the population without chronic cough, the odds ratios (ORs) were analyzed to evaluate the impact of these factors on chronic cough. In multivariable analysis, only current smoking status, UACS, COPD, asthma, and abnormal chest radiographs were independently associated with chronic cough, and the adjusted ORs were 3.16, 2.50, 2.41, 8.89, and 2.74, respectively (Table 4). Current smoking, UACS, COPD, asthma, and abnormal chest radiographs were sequentially observed in subjects with chronic cough; however, the impact of chronic cough was strongest for asthma, followed in descending order by current smoking status, chest radiographic abnormality, UACS, and COPD.

**Table 4:**
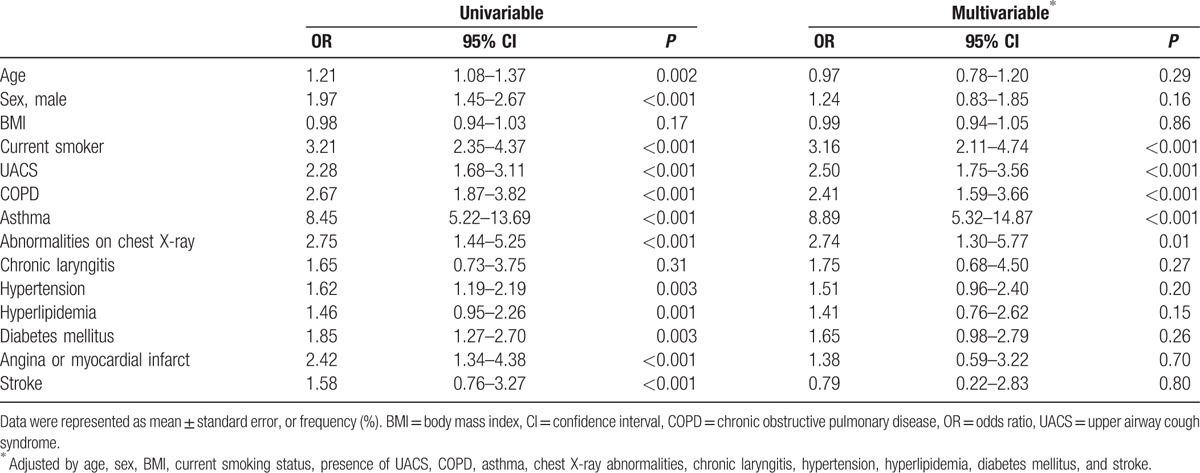
Risk factors contributing to chronic cough in univariable and multivariable analysis.

### 3.4 Comparison according to possible causes

We compared the clinical characteristics of the subjects according to current smoking status, UACS, COPD, asthma, and abnormal chest radiographs to determine whether associated symptoms could be used to differentiate causes of chronic cough. The most frequently associated symptom was phlegm (75.9% ± 3.0%). All groups showed a high frequency of chronic sputum production, but the frequency was significantly higher in current smokers (86.9% ± 3.7%). The UACS group reported more episodes of dyspnea and night sweats, and the COPD group reported more occurrences of blood-tinged sputum. However, there was no specific clinical characteristic suggesting asthma or chest radiographic abnormality (Table 5). When clinical characteristics were compared between subjects with a single cause and those with 2 or more causes, subjects with multiple causes complained more frequently of dyspnea and weight loss (Table S2).

**Table 5:**
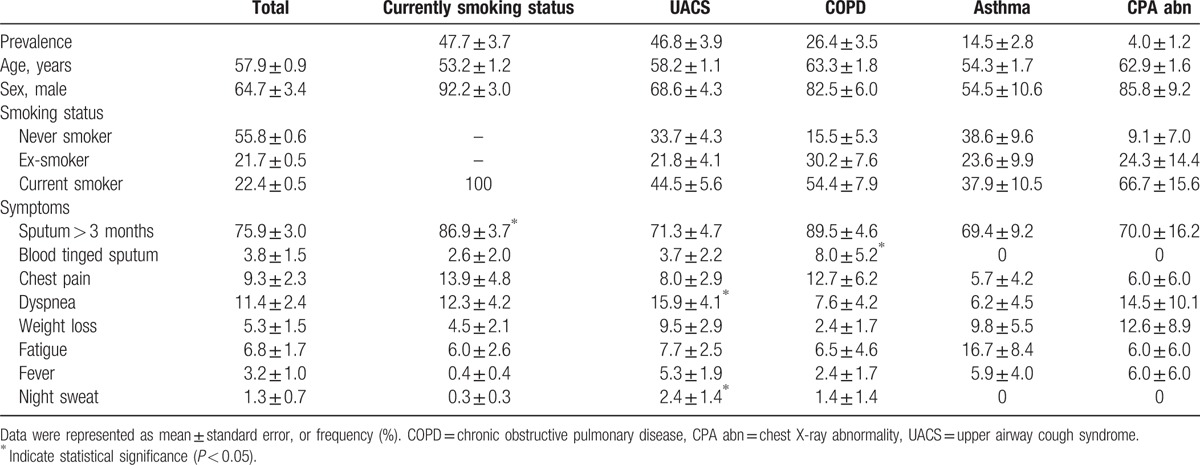
Comparing clinical characteristics between possible causes of chronic cough.

## 4 Discussion

Based on nationwide survey, we identified the prevalence of chronic cough in the Korean population and analyzed the prevalence of possible contributors.

To the best of our knowledge, our results are derived from a larger population including smokers than in previous reports^[25–35]^ and ours is the first report on the prevalence of chronic cough in the Korean population. We found the overall prevalence of chronic cough in the general adult Korean population to be 2.5% ± 0.2%, and the common possible causes, in descending order, to be current smoking, UACS, COPD, asthma, and chest radiographic abnormalities. This pattern was not different according to smoking status, although COPD was more prevalent in cigarette smokers.

Individual causes of chronic cough may overlap. In this analysis, UACS frequently accompanied asthma, and chest radiographic abnormalities were frequently observed in subjects with both COPD and asthma. In multivariable analysis, the conditions that cause chronic cough were asthma, current smoking, chest radiographic abnormality, UACS, and COPD, in descending order. More frequently associated symptoms were chronic phlegm for current smokers, dyspnea and night sweats for those with UACS, and blood-tinged sputum for those with COPD.

We believe that our study have some merits and could contribute more to clinical practice. First, previous studies of causes of chronic cough were performed in a relatively small number of patients seen in a specific clinic, so risked selection bias. In this study, although the number of patients with chronic cough may seem small, the survey population selected is regarded as representative of the Korean general population. Second, most of the previous studies excluded smokers or patients with abnormal chest radiographs, and clinicians have had questions about the general frequency of chest radiographic abnormalities and the different characteristics of smokers. Our data showed not only a high prevalence of cigarette smoking (47.7%) in subjects with chronic cough, but also a strong impact of smoking (OR 3.16) on the prevalence of chronic cough independent of underlying diseases. Third, our study found that the prevalence of abnormal chest radiographs, which could be a cause of chronic cough, is 4.0% in the general population. However, the OR for chronic cough was higher than for COPD or UACS, underscoring the importance of chest radiography, especially in a region with a high prevalence of tuberculosis.

Unlike previous research, which reported the prevalence of chronic bronchitis to be 5%,^[7]^ our study reported the prevalence of COPD to be as high as 26.4%. In a previous report, the overall prevalence of COPD in Koreans older than 40 years was 13.4% and increased to 19.4% in men.^[36]^ In our analysis, 12.2% of the total study population had COPD. Focusing on patients with chronic cough, the prevalence of COPD increased to 26.4%. Therefore, physicians should not underestimate the possibility of COPD in patients with chronic cough.

A diagnosis of GERD needs to be confirmed with 24-hour pH monitoring, so the KNHANES data could not show accurately the prevalence or clinical impact of this condition. However, GERD has been reported to be the 3rd most common cause of chronic cough and its frequency is estimated to be 10% to 21%.^[7,37]^ Otorhinolaryngologic examinations cannot completely replace pH monitoring for diagnosing GERD; however, we used reflux laryngitis as a clue for determining the presence of GERD. Surprizingly, the prevalence of chronic laryngitis was low (4.1% ± 1.6%) and not significantly different from the prevalence in the population without chronic cough (2.7% ± 0.3%). This may imply the fact that GERD-related cough may not be prevalent in the Korean population. Alternatively, the prevalence of GERD in Asian patients may be different from that in previous reports on Western patients with chronic cough.^[38]^ Our data may highlight a need for different algorithms to establish the etiology of chronic cough in different races.

Despite the interesting findings of this study, there are several potential limitations. First, as this study was a cross-sectional analysis, each cause was not confirmed by response to treatment. However, chronic cough is widely known to have multiple causes, and the relative risk for each of these was calculated. Describing the pattern of presentation and considering all possible causes in the general population would be meaningful for physicians encountering patients with chronic cough. Second, the prevalence of asthma was calculated using a self-reported questionnaire and not by a provocation test, so there is a risk of classifying a patient as false positive or as false negative. Although we categorized patients as having asthma diagnosed by their doctor, we acknowledge the possibility of misdiagnosis or underdiagnosis, and there is limitation in differentiating COPD from asthma or asthma-COPD overlap syndrome. Third, we used the prevalence of chronic laryngitis instead of pH monitoring to diagnose. GERD frequently leads to chronic laryngitis;^[39–42]^ however, there are some differences in the mechanism of diseases, and these changes may also appear secondary to smoking, excessive alcohol, allergies, asthma, or voice abuse.^[37,42]^ Fourth, medication effects, such as those of angiotensin-converting enzyme inhibitors, were not evaluated since most participants did not know the exact name of their prescribed medication. Therefore, we could not determine the prevalence of angiotensin-converting enzyme inhibitor-related cough. Finally, we did not identify other diseases causing chronic cough that could be diagnosed by other methods, such as sputum analysis, computed tomography, or bronchoscopy. Therefore, the number of patients who cannot find the cause of cough may be overestimated.

In conclusion, GERD-related cough is not prevalent in Korean population, and more attention should be paid to smoking and COPD in subjects with chronic cough along with asthma or UACS. Further effort to develop a protocol for chronic cough is necessary for Asian populations.

## Supplementary Material

Supplemental Digital Content

## Supplementary Material

Supplemental Digital Content
