# Supplementary material for: Prevalence of chronic cough and possible causes in the general population based on the Korean National Health and Nutrition Examination Survey
Source: Medicine (Baltimore). 2016 Sep 16;95(37):e4595. doi: 10.1097/MD.0000000000004595 (PMC5402551; doi:10.1097/MD.0000000000004595)
Supplement: Supplemental Digital Content [file medi-95-e4595-s001.pdf]

## Supplementary tables

Table S1. Clinical characteristics of the cases with chronic cough according to smoking status

| | Total | | | | |
|---|---|---|---|---|---|
| | | Never smoker | Ex-smoker | Current smoker | *P*-value* |
| Prevalence, % | | 32.0 ± 3.2 | 20.3 ± 2.9 | **47.7** ± 3.8 | |
| Age, year | 58.0 ± 0.9 | 60.9 ± 1.5 | 62.3 ± 2.0 | 53.2 ± 1.2 | 0.01 |
| Sex, male | 64.7 ± 3.5 | 8.0 ± 3.2 | 88.9 ± 4.3 | 92.2 ± 3.0 | <0.001 |
| BMI, kg/m$^2$ | 23.9 ± 0.2 | 24.0 ± 0.3 | 23.6 ± 0.5 | 24.0 ± 0.4 | 0.73 |
| Underlying diseases | | | | | |
| Hypertension | 36.9 ± 3.5 | 42.3 ± 5.9 | 47.4 ± 8.3 | 29.7 ± 5.2 | 0.32 |
| Hyperlipidemia | 18.4 ± 3.3 | 20.3 ± 5.1 | 27.2 ± 7.2 | 14.4 ± 5.4 | 0.75 |
| Diabetes mellitus | 15.4 ± 2.5 | 14.8 ± 4.3 | 25.1 ± 7.0 | 14.8 ± 3.9 | 0.60 |
| History of tuberculosis | 15.8 ± 2.7 | 14.9 ± 4.3 | 24.4 ± 7.2 | 12.9± 4.2 | 0.80 |
| Angina, MI | 7.1 ± 1.9 | 6.2 ± 2.5 | 10.1 ± 4.9 | 4.0 ± 2.4 | 0.91 |
| Stroke | 3.0 ± 1.1 | 4.7 ± 2.8 | 5.9 ± 2.6 | 0.9 ± 0.6 | 0.36 |
| Findings on Chest X-ray | | | | | 0.18 |
| Normal | 72.2 ± 3.4 | 77.8 ± 5.5 | 56.4 ± 8.7 | 78.6 ± 4.6 | |
| Inactive lesion | 23.7 ± 3.1 | 21.1 ± 5.5 | 38.8 ± 8.5 | 16.1 ± 3.8 | |
| Active lesion | 4.1 ± 1.2 | 1.1 ± 0.8 | 4.8 ± 3.2 | 5.4 ± 2.3 | |
| Number of causes | | | | | <0.001 |
| Unidentified | 14.7 ± 3.1 | 38.1 ± 7.3 | 15.9 ± 5.5 | 0 | |
| 1 | 35.0 ± 4.4 | 37.1 ± 6.7 | 54.8 ± 9.8 | 25.8 ± 6.0 | |
| 2 | 37.6 ± 4.1 | 24.8 ± 6.5 | 29.2 ± 8.7 | 48.8 ± 6.1 | |
| 3 | 9.7 ± 2.9 | | | 19.5 ± 5.4 | |
| 4 | 2.9 ± 1.6 | | | 5.9 ± 3.3 | |

Data were represented as mean ± standard error, or frequency (%)

Abbreviation: BMI, body mass index; COPD, chronic obstructive pulmonary disease; UACS, upper airway cough syndrome; TB, tuberculosis; EQ5D, EuroQOL instrument
