# Supplementary material for: Prevalence of chronic cough and possible causes in the general population based on the Korean National Health and Nutrition Examination Survey
Source: Medicine (Baltimore). 2016 Sep 16;95(37):e4595. doi: 10.1097/MD.0000000000004595 (PMC5402551; doi:10.1097/MD.0000000000004595)
Supplement: Supplemental Digital Content [file medi-95-e4595-s002.pdf]

Table S2. Clinical characteristics of patients with multiple possible causes of chronic cough

Symptoms	Chronic cough with single cause	Chronic cough with multiple causes	*P*-value
Prevalence	49.7 ± 4.5	50.3 ± 4.5	
Sputum > 3 months	76.5 ± 4.2	79.5 ± 5.2	0.66
Blood tinged sputum	1.3 ± 1.3	2.6 ± 1.8	0.56
Chest pain	7.0 ± 3.0	11.5 ± 4.2	0.39
Dyspnea	1.6 ± 0.9	12.9 ± 4.6	0.001*
Weight loss	0.7 ± 0.6	6.5 ± 2.8	0.04*
Fatigue	3.0 ± 1.6	9.5 ± 3.7	0.08
Fever	0.8 ± 0.6	4.3 ± 2.5	0.08
Night sweat	1.6 ± 1.2	1.5 ± 1.5	0.95
EQ5D-index	0.91 ± 0.02	0.91 ± 0.02	0.73
EQ5D-VAS	70.4 ± 2.6	67.2 ± 2.4	0.39

Abbreviation: EQ5D, EuroQOL instrument
